# Evaluation of gliomas peritumoral diffusion and prediction of IDH1 mutation by IVIM-DWI

**DOI:** 10.18632/aging.202751

**Published:** 2021-03-26

**Authors:** Taifu Gu, Ting Yang, Jianglong Huang, Jianhua Yu, Hongxin Ying, Xinlan Xiao

**Affiliations:** 1 Medical Imaging Center, The Second Affiliated Hospital of Nanchang University, Nanchang, Jiangxi Province, China; 2 Department of Radiology, The First Affiliated Hospital of Medical College, Zhejiang University, Hangzhou, Zhejiang Province, China

**Keywords:** IVIM-DWI, 3D pCASL, glioma, IDH1 mutation

## Abstract

Glioma characterized by high morbidity and mortality, is one of the most common brain tumors. The application of intravoxel incoherent motion diffusion weighted imaging (IVIM-DWI) in differentiating glioma grading and IDH1 mutation status were poorly investigated. 78 glioma patients confirmed by pathological and imaging methods were enrolled. Glioma patients were measured using IVIM-DWI, then related parameters such as cerebral blood flow (CBF), perfusion fraction (f), pseudo diffusivity (D*), and true diffusivity (D), were derived. Receiver operating characteristic (ROC) curves were made to calculate specificity and sensitivity. The values of CBF1, CBF3, D*1, rCBF1-2, rCBF3-2, and age in group high-grade gliomas (HGG) were significantly higher than that of in group low-grade gliomas (LGG). The values of CBF1, CBF3, rCBF1-2, rCBF3-2, D*1, and age in group IDH1^*mut*^ were significantly lower than that of in group IDH1^*wt*^. The levels of D1 and f1 were remarkably higher in the group IDH1^*mut*^ than group IDH1^*wt*^. rCBF1-2 had a remarkably positive correlation with CBF1 (r=0.852, p<0.001). f1 showed a markedly negative correlation with CBF1 (r= -0.306, p=0.007). IVIM-DWI presented efficacy in differentiating glioma grading and IDH1 mutation status.

## INTRODUCTION

Glioma, originated from neuroepithelial cells, is the most common central nervous system tumor. The prognosis of glioma patients was affected deeply by tumor resection. Glioma recurrence usually occurs in the area around the tumor residual cavity [1–3]. It is difficult to distinguish the boundary between glioma and normal brain tissue by naked eyes during operation. Therefore, definition of tumor boundary is very important [4]. The tumor tissues should be removed as much as possible under the premise of avoiding damage of normal brain tissue.

In the past decades, medical imaging technology has been developed greatly. Medical imaging technology plays a key role in determining tumor boundary, observing tumor resection dynamically, and achieving precise positioning for biopsy and radiotherapy of target areas [5]. Currently, the most widely used imaging technology in neuronavigation is magnetic resonance imaging (MRI). T1 enhanced scan is often used as the imaging basis for neuronavigation resection of glioma. However, most of the low-grade gliomas (LGG) and 10% - 30% of the high-grade gliomas (HGG) have no enhancement or only a little enhancement due to the fact that blood-brain barrier is not completely destroyed [6]. It is difficult to determine the histological margin of the tumor only based on T1WI (T1 weighted imaging) enhancement. T2WI (T2 weighted imaging) is usually used to determine the tumor boundary, and it can well display peritumoral edema [7]. However, it is uncertain whether there must be tumor cell infiltration in the peritumoral edema area, which may lead to over resection of normal brain tissue [8]. Therefore, conventional MRI method have limitation in defining tumor boundaries, and new imaging techniques (especially functional imaging) need to be further explored to accurately define tumor histological margin.

Arterial water was used as an endogenous tracer in three-dimensional pseudo-continuous arterial spin labeling (3D pCASL), which has been widely applied in disease diagnose and treatment [9]. Cerebral blood flow (CBF) could be detected using 3D pCASL. However, 3D pCASL might be limited by transit time, and the tumor slow blood flowing might be underestimated by 3D pCASL due to tortuous vessels.

Intravoxel incoherent motion diffusion weighted imaging (IVIM-DWI) diffusion imaging is one of the most popular functional MRI imaging techniques in recent years [10]. The diffusion coefficient of continuous distribution of micro motion in voxel is assumed to be two parts, namely D (diffusion coefficient) and D * (pseudo-diffusion coefficient), which represent slow and fast diffusion, respectively. f is the perfusion fraction representing the volume ratio of perfusion effect produced by local microcirculation to the total volume [11, 12]. At present, IVIM-DWI has been widely used in differential diagnosis, grading, and prediction of survival time and prognosis of brain tumors [13–15]. However, the application of IVIM-DWI and 3D pCASL on gliomas grading has not been well elucidated.

Isocitrate dehydrogenase 1 (IDH 1) catalyzes the production of alpha-ketoglutarate, and further increase the level of nicotinamide adenine dinucleotide phosphate (NADPH). NADPH plays a key role regulating cell redox system. IDH1 mutation (IDH1^*mut*^) may lead to the increase of oxidative stress level [16]. It was reported that IDH1^*mut*^ was found in the 70% LGG patients [17, 18]. In addition, seizure was more likely to occur in the gliomas patients with IDH1^*mut*^ than IDH1 wild-type (IDH1^*wt*^) patients [19]. Therefore, the identification of IDH1 gene status in gliomas patients might be helpful for tumor grading and further treatment. Currently, systemically analysis of IDH1 in gliomas patients using IVIM has not been reported.

In this study, the parameters derived from IVIM-DWI between group HGG and LGG, group IDH1^*mut*^ and IDH1^*wt*^ were analyzed. ROC analysis of IVIM-DWI-derived parameters in gliomas grade and IDH1 mutation status was analyzed. This study may provide evidence for the application of IVIM-DWI and 3D pCASL in glioma grading and IDH1 mutation predication.

## RESULTS

### Patients characteristics

**Figure 1:**
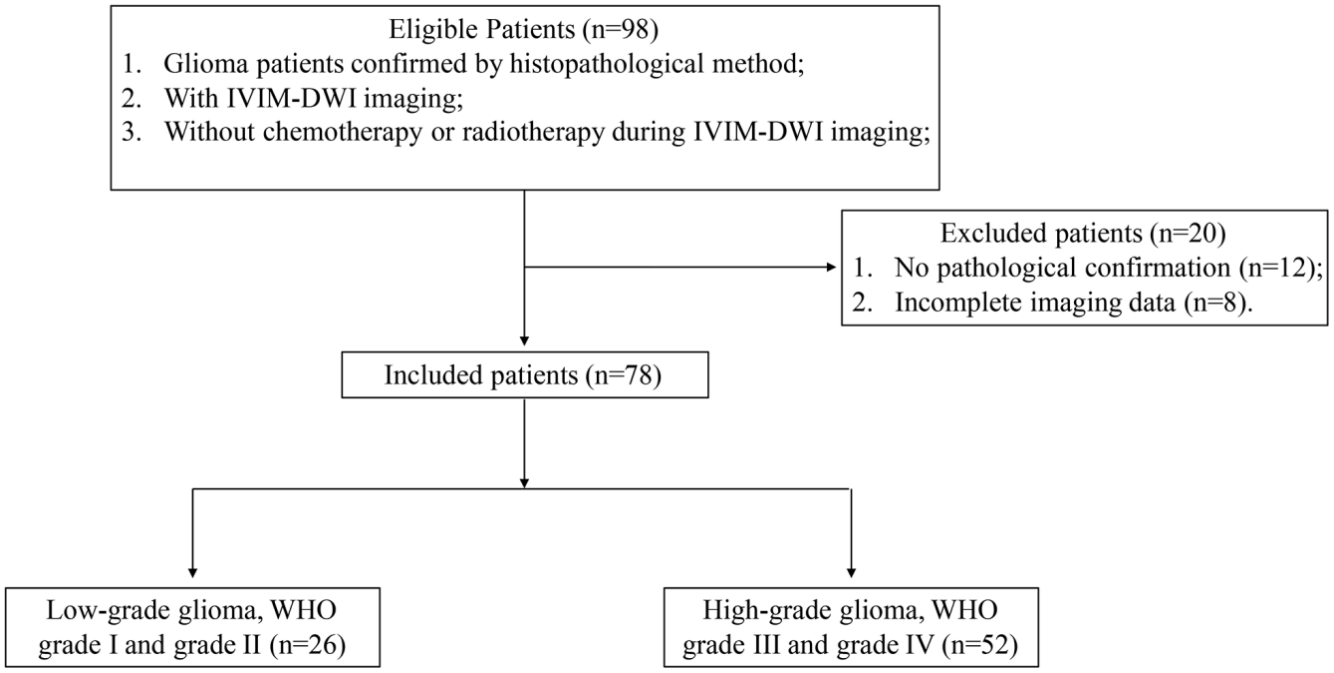
**Flowchart of patient exclusion and inclusion criteria.**

A total of 78 patients (males: 46, female: 32, age range: 13-76, average age of males: 49±12, average age of females: 50±15) were enrolled in this research (Figure 1). Totally, 26 patients were diagnosed as WHO grade I and grade II (males:16, females: 10, IDH1+: 16, IDH1-: 10, age range: 14-65), 52 cases were diagnosed as WHO grade III and grade IV (males:30, females: 22, IDH1+: 11, IDH1-: 41, age range: 13-76).

### Interobserver agreement

Good interobserver agreement in terms of D* (ICC: 0.978, 95% CI: 0.916-0.948), D (ICC: 0.892, 95% CI: 0.826-0.922), f (ICC: 0.934, 95% CI: 0.864-0.896), and CBF (ICC: 0.884, 95% CI: 0.916-0.935) were observed. Meanwhile, the coefficients of variations of IVIM-derived parameters ranged from 6.5-15.8%.

### Comparison of parameter values between group HGG and LGG

**Figure 2:**
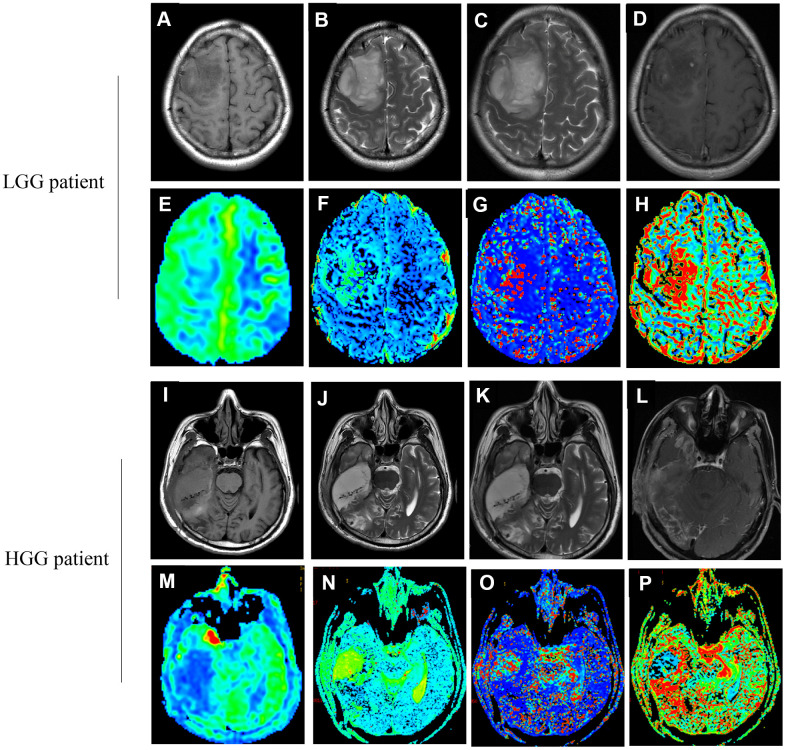
Images (**A**–**H**) correspond to a 37-year old female with LGG (WHO grade II, IDH1mut). Images (**I**–**P**) correspond to a 47-year old male with HGG (WHO grade IV, IDH1wt). (**A**, **I**) The T1 image; (**B**, **J**) The T2 image; (**C**, **K**) The T2WI image; (**D**, **L**) The T1WI+C image; (**E**, **M**) The CBF map; (**F**, **N**) The D map; (**G**, **O**) The D* map; (**H**, **P**) The f map. (true diffusion coefficient: (**D**) perfusion-related diffusion coefficient: D*; perfusion fraction: f; cerebral blood flow: CBF).

Remarkable differences were observed in some parameters of IVIM perfusion between group HGG and LGG (Table 1 and Figure 2). The values of CBF1 (141.69±72.77 vs 75.93±66.81 ml/min/100g), CBF3 (53.13±10.05 vs 42.94±11.78 ml/min/100g), D*1 (57.69±35.49 vs 49.9±30.38 ×10^-3^mm^2^/sec), and age (53.79±13.2 vs 41.92±13.93 years) in group HHG were significantly higher than that of in group LGG. The D1 value related with diffusion was remarkably lower in group HGG than group LGG (0.506±0.34 vs 0.715±0.325 ×10^-3^mm^2^/sec). The values of CBF1 and CBF3 were normalized to the value of CBF2 representing the mirror side normal areas, then the values of rCBF1-2 and rCBF3-2 were achieved. The values of rCBF1-2 and rCBF3-2 in group HGG were markedly higher than group LGG (Table 1). Therefore, remarkable difference could be observed these parameters such as CBF1, CBF3, rCBF1-2, rCBF3-2, D1, and D*1 between group HGG and LGG using IVIM-DWI. No significant difference was found in terms of gender between group HGG and LGG.

**Table 1:** Comparison of parameter values between group HGG and LGG.

**Parameters**	**HGG (n=52)**	**LGG (n=26)**	**P-value**
Age (years)	53.79±13.2	41.92±13.93	<0.001
Gender	Male:30, Female:22	Male:16, Female:10	0.745
CBF1 (ml/min/100g)	141.69±72.77	75.93±66.81	<0.001
CBF2 (ml/min/100g)	44.816±14.142 *	50.334±10.635 *	0.083
CBF3 (ml/min/100g)	53.13±10.05 *	42.94±11.78 *	<0.001
rCBF1-2 (ml/min/100g)	3.34±1.79	1.56±1.45	<0.001
rCBF3-2 (ml/min/100g)	1.36±0.73	0.88±0.25	0.002
D1 (×10^-3^mm^2^/sec)	0.506±0.34	0.715±0.325	0.012
D*1 (×10^-3^mm^2^/sec)	57.69±15.49	49.9±10.38	0.013
f1	0.389±0.168	0.394±0.204	0.850
D2 (×10^-3^mm^2^/sec)	0.369±0.244 **	0.373±0.192 **	0.946
D*2 (×10^-3^mm^2^/sec)	38.2±28.4 ***	43.6±39.6 ***	0.491
f2	0.329±0.1146	0.315±0.089	0.591
D3 (×10^-3^mm^2^/sec)	0.29±0.226 **	0.3446±0.2336 **	0.358
D*3 (×10^-3^mm^2^/sec)	72.1±58 ***	65.4±27.8 ***	0.584
f3	0.405±0.125	0.412±0.128	0.802

*<0.05 compared with CBF1, **<0.05 compared with D1, ***<0.05 compared with D*1.

In addition, the differences of parameters derived using IVIM in tumor parenchyma, peritumoral, and mirror side normal areas were also analyzed (Table 1). In the group HGG, significant higher values of CBF and D in tumor parenchyma areas were found compared with peritumoral, and mirror side normal areas. Meanwhile, remarkable difference in terms of D* was observed between tumor areas and other regions. Similar findings were observed in the group LGG (Table 1). These results indicate that the difference of gliomas grade and tumor location could be effectively achieved using IVIM-DWI.

### Comparison of parameter values between group IDH1^*mut*^ and IDH1^*wt*^

The gene mutation status of IDH1 in all patients were measured using immunohistochemical method firstly. Then, IVIM derived parameters between group IDH1+ and IDH1- were analyzed (Table 2). The values of CBF1 (100.127±53.092 vs 150.172±35.020 ml/min/100g), CBF3 (46.197±13.134 vs 51.603±10.408 ml/min/100g), rCBF1-2 (2.049±1.879 vs 3.116±1.785 ml/min/100g), rCBF3-2 (1.038±0.567 vs 1.579±0.566 ml/min/100g), D*1 (50.9±16 vs 59.65±13 ×10^-3^mm^2^/sec), and age (45.22±10.297 vs 52.27±15.837 years) in group IDH1^*mut*^ were significantly lower than that of in group IDH1^*wt*^ (Table 2). However, the levels of D1 (0.635±0.206 vs 0.635±0.206 ×10^-3^mm^2^/sec) and f1 (0.449±0.206 vs 0.356±0.156) were remarkably higher in the group IDH1^*mut*^ than group IDH1^*wt*^. No significant difference was found in terms of gender between group IDH1^*mut*^ and IDH1^*wt*^.

**Table 2:** Comparison of parameter values between group IDH1^*mut*^ and IDH1^*wt*^.

**Parameters**	**IDH1^*mut*^ (n=27)**	**IDH1^*wt*^ (n=51)**	**P-value**
Age (years)	45.22±10.297	52.27±15.837	0.04
Gender	Male:16, Female:11	Male:30, Female:21	0.97
CBF1 (ml/min/100g)	100.127±53.092	150.172±35.020	0.005
CBF2 (ml/min/100g)	50.236±12.383 *	44.7598±13.446 *	0.083
CBF3 (ml/min/100g)	46.197±13.134 *	51.603±10.408 *	0.02
rCBF1-2 (ml/min/100g)	2.049±1.879	3.116±1.785	0.016
rCBF3-2 (ml/min/100g)	1.038±0.567	1.579±0.566	0.019
D1 (×10^-3^mm^2^/sec)	0.635±0.206	0.3445±0.166	0.037
D*1 (×10^-3^mm^2^/sec)	50.9±16	59.65±13	0.04
f1	0.449±0.206	0.356±0.156	0.029
D2 (×10^-3^mm^2^/sec)	0.329±0.18 **	0.392±0.2465 **	0.241
D*2 (×10^-3^mm^2^/sec)	36±39.7 ***	42±28 ***	0.434
f2	0.356±0.096	0.308±0.11	0.057
D3 (×10^-3^mm^2^/sec)	0.306±0.23 **	0.313±0.229 **	0.893
D*3 (×10^-3^mm^2^/sec)	62±35 ***	73.9±56.1 ***	0.329
f3	0.445±0.115	0.389±0.127	0.056

*<0.05 compared with CBF1, **<0.05 compared with D1, ***<0.05 compared with D*1.

Meanwhile, the differences of parameters in tumor parenchyma, peritumoral, and mirror side normal areas were also analyzed in both group IDH1^*mut*^ and IDH1^*wt*^. Significant differences in values of CBF, D, and D* could be found between tumor areas and other regions (peritumoral and mirror side normal areas). Therefore, IVIM-DWI might be a promising method to predict IDH1 gene mutation and evaluation of gliomas peritumoral diffusion.

In addition, the cases distribution in the group HGG, LGG, IDH1+, IDH1^*wt*^ were analyzed (Table 3). Totally, 41 cases IDH1^*wt*^ patients were found in the group HGG, only 10 cases IDH1^*wt*^ patients in the group LGG. Meanwhile, 11 and 16 cases IDH1^*mut*^ patients were found in the group HGG and LGG, respectively (Table 3). The distribution difference was significant indicating that IDH1 wide type gliomas patients were more likely to be diagnosed as HGG.

**Table 3:** Statistic analysis of IDH1^*mut*^ and IDH1^*wt*^ cases in the group HGG and LGG.

**Items**	**HGG (n=52)**	**LGG (n=26)**	**x^2^**	**P-value**
IDH1^*mut*^	11 (14.1%)	16 (20.5%)	12.49	<0.001
IDH1^*wt*^	41 (52.6%)	10 (12.8%)	

### ROC analysis of IVIM-DWI-derived parameters in IDH1 mutation and gliomas grade

**Figure 3:**
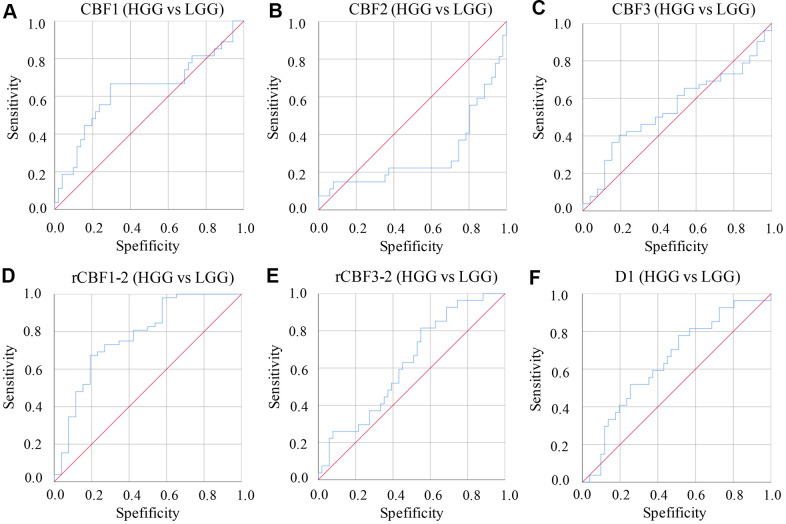
**ROC curves of parameters derived from IVIM-DWI between group LGG and HGG.** (**A**) ROC of CBF1 in differentiating LGG and HGG; (**B**) ROC of CBF2 in differentiating LGG and HGG; (**C**) ROC of CBF3 in differentiating LGG and HGG; (**D**) ROC of rCBF1-2 in differentiating LGG and HGG; (**E**) ROC of rCBF3-2 in differentiating LGG and HGG; (**F**) ROC of D1 in differentiating LGG and HGG. (cerebral blood flow: CBF; the CBF of tumor parenchyma areas: CBF1; the CBF of mirror side normal areas: CBF2; the CBF of peritumoral areas: CBF3; rCBF1-2= CBF1/CBF2; rCBF3-2= CBF3/CBF2).

The AUC values of parameters derived from IVIM-DWI were calculated between group HGG and LGG (Table 4 and Figure 3). The AUC value of rCBF1-2 was the highest, 0.861 (P<0.001) with specificity 94.1% and sensitivity 92.3%. Relative high AUC values were also observed in CBF1 (0.818), CBF3 (0.774), rCBF3-2 (0.773), and D1 (0.816). High specificity and sensitivity were observed in CBF1 (specificity: 82.4%, sensitivity: 88.5%) and D1 (specificity: 86.9%, sensitivity: 81.2%) (Table 4 and Figure 3).

**Table 4:** Identification of HGG and LGG by IVIM-DWI-derived parameters.

**Parameters**	**AUC**	**Sensitivity (%)**	**Specificity (%)**	**P-value**
CBF1 (ml/min/100g)	0.818	88.5	82.4	0.072
CBF2 (ml/min/100g)	0.365	11.5	3.8	0.054
CBF3 (ml/min/100g)	0.774	67.3	74.8	0.062
rCBF1-2 (ml/min/100g)	0.861	92.3	94.1	<0.001
rCBF3-2 (ml/min/100g)	0.773	69.2	69.8	0.082
D1 (×10^-3^mm^2^/sec)	0.816	81.2	86.9	0.068
D*1 (×10^-3^mm^2^/sec)	0.398	56.6	46.6	0.142
f1	0.498	19.8	76.8	0.975
D2 (×10^-3^mm^2^/sec)	0.487	23.1	11.5	0.853
D*2 (×10^-3^mm^2^/sec)	0.455	5.8	3.8	0.521
f2	0.547	40.4	19.6	0.498
D3 (×10^-3^mm^2^/sec)	0.849	84.6	82.2	0.045
D*3 (×10^-3^mm^2^/sec)	0.484	28.8	18.4	0.82
f3	0.461	75	76.4	0.578

**Figure 4:**
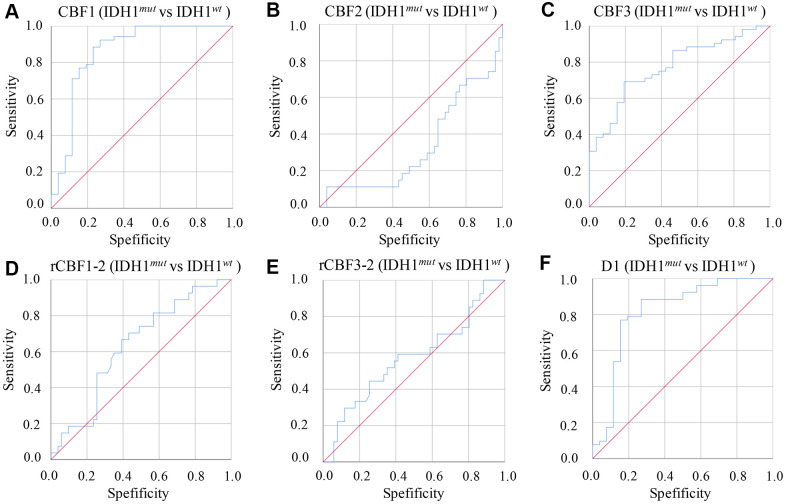
**ROC curves of parameters derived from IVIM-DWI between group IDH1^*mut*^ and IDH1^*wt*^.** (**A**) ROC of CBF1 in differentiating IDH1^*mut*^ and IDH1^*wt*^; (**B**) ROC of CBF2 in differentiating IDH1^*mut*^ and IDH1^*wt*^; (**C**) ROC of CBF3 in differentiating IDH1^*mut*^ and IDH1^*wt*^; (**D**) ROC of rCBF1-2 in differentiating IDH1^*mut*^ and IDH1^*wt*^; (**E**) ROC of rCBF3-2 in differentiating IDH1^*mut*^ and IDH1^*wt*^; (**F**) ROC of D1 in differentiating IDH1^*mut*^ and IDH1^*wt*^. (cerebral blood flow: CBF; the CBF of tumor parenchyma areas: CBF1; the CBF of mirror side normal areas: CBF2; the CBF of peritumoral areas: CBF3; rCBF1-2= CBF1/CBF2; rCBF3-2= CBF3/CBF2).

The diagnosing efficiency using IDH1^*mut*^ and IDH1^*wt*^ was also calculated by analyzing parameters derived from IVIM-DWI (Table 5 and Figure 4). The CBF1 presented the highest ADU value, 0.892 (P=0.003), with specificity 82% and sensitivity 82.4%. Relative high ADU values for D1 (0.873, P<0.001) and CBF3 (0.831, P=0.002) were also found. The D1 (specificity: 85.5%, sensitivity: 84.4%) and CBF3 (specificity: 83.9%, sensitivity: 81.1%) showed good diagnosing efficiency (Table 5 and Figure 4).

**Table 5:** Identification of IDH1^*mut*^ and IDH1^*wt*^ by IVIM-DWI-derived parameters.

**Parameters**	**AUC**	**Sensitivity (%)**	**Specificity (%)**	**P-value**
CBF1 (ml/min/100g)	0.892	82.4	82	0.003
CBF2 (ml/min/100g)	0.442	47.8	51	0.515
CBF3 (ml/min/100g)	0.831	81.1	83.9	0.002
rCBF1-2 (ml/min/100g)	0.775	77.4	82	<0.001
rCBF3-2 (ml/min/100g)	0.791	73.7	86.6	0.003
D1 (×10^-3^mm^2^/sec)	0.873	84.4	85.5	<0.001
D*1 (×10^-3^mm^2^/sec)	0.501	23.2	22.8	0.992
f1	0.633	66.7	29.3	0.054
D2 (×10^-3^mm^2^/sec)	0.419	96.3	86.3	0.242
D*2 (×10^-3^mm^2^/sec)	0.365	98.4	96.1	0.051
f2	0.624	81.5	54.9	0.073
D3 (×10^-3^mm^2^/sec)	0.517	40.7	27.5	0.805
D*3 (×10^-3^mm^2^/sec)	0.45	96.3	88.2	0.469
f3	0.631	76.4	39.2	0.058

### Correlation analysis of IVIM-DWI-derived parameters and CBF1

**Figure 5:**
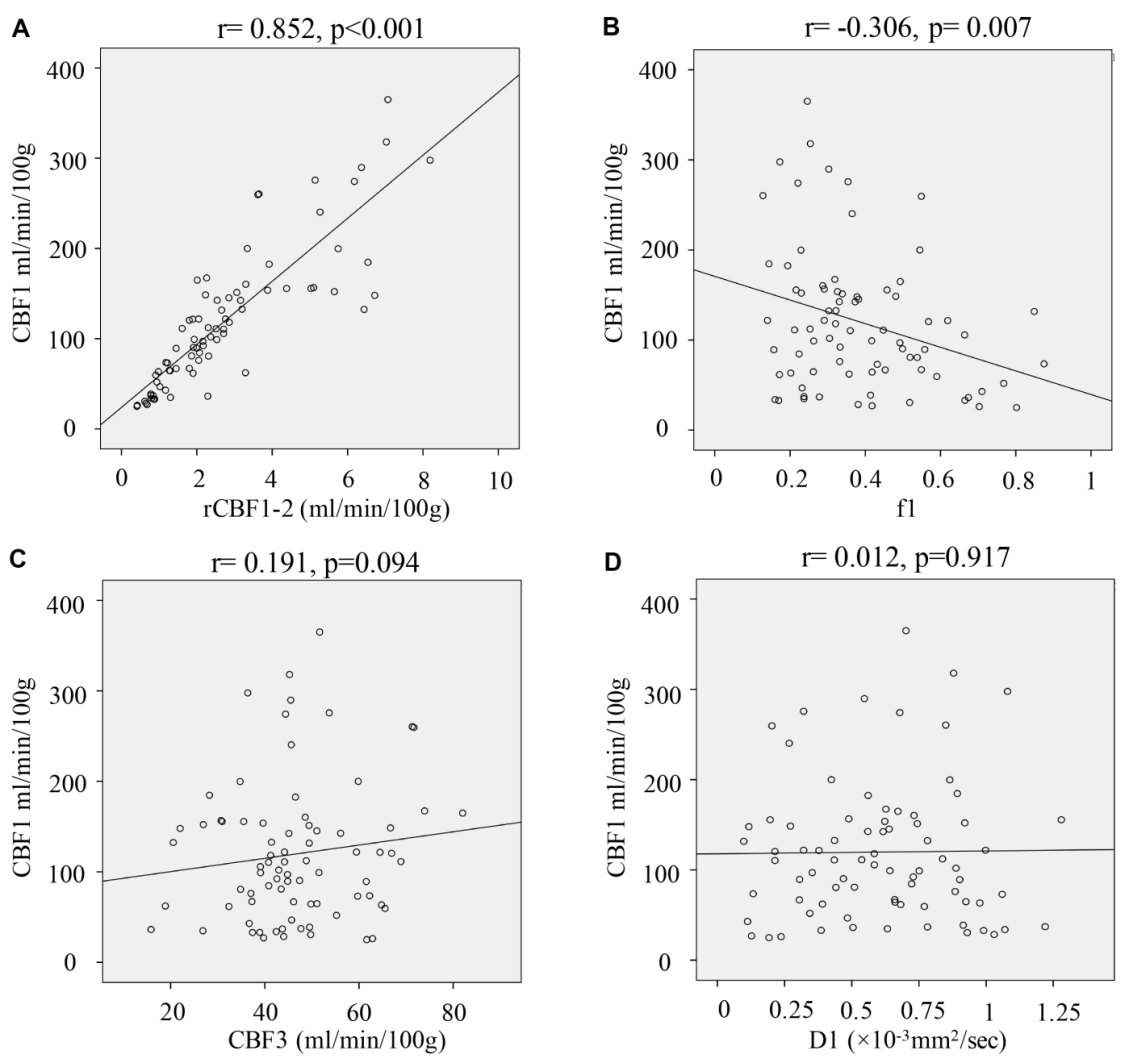
**Correlation analysis of IVIM-DWI-derived parameters and CBF1.** (**A**) Correlation analysis of rCBF1-2 and CBF1; (**B**) Correlation analysis of f1 and CBF1; (**C**) Correlation analysis of CBF3 and CBF1; (**D**) Correlation analysis of D1 and CBF1. (cerebral blood flow: CBF; the CBF of tumor parenchyma areas: CBF1; the CBF of mirror side normal areas: CBF2; the CBF of peritumoral areas: CBF3; rCBF1-2= CBF1/CBF2; rCBF3-2= CBF3/CBF2).

The correlation relationship between CBF1 other IVIM-DWI-derived parameters were analyzed using Pearson method (Figure 5). rCBF1-2 (Figure 5A) had a remarkably positive correlation with CBF1 (r=0.852, p<0.001), and f1 (Figure 5B) showed a markedly negative correlation with CBF1 (r= -0.306, p=0.007). However, other parameters including CBF3 (Figure 5C) and D1 (Figure 5D) did not present remarkable correlation with CBF1.

## DISCUSSION

Perfusion has been proved to increase the diagnose sensitivity of many diseases including glioma. IVIM-DWI and 3D pCASL have been believed to be repeatable, safe, effective, and convenient to diagnose and grade brain tumor including glioma though detecting blood supply [20].

There are many different kinds of parameter maps in IVIM images. Previous study showed that the boundaries of tumor, peritumoral and normal brain tissues were clear defined by D, D*, and f [21]. Therefore, the most valuable parameters for delineation of tumor boundary were selected to guide clinical diagnosis and treatment. Some parameters derived from IVIM-DWI such as D*, f, and CBF have been widely used in the fields of disease predication, diagnose, and treatments [20, 22]. D* could represent the incoherent movement of blood in the microvasculature compartment. In addition, the ratio of incoherent signal arising from vascular compartment could be represented by f. Previous report indicated that IVIM derived CBF and f agree reasonably with traditional cerebral blood volume (CBV) from dynamic susceptibility contrast (DSC) [23]. CBF, D, rCBF1-2, and D*1 were effective parameters to distinguish HGG and LGG (Table 1). Meanwhile, IDH1^*mut*^ and IDH1^*wt*^ could be differentiated with CBF, rCBF1-2, D1, D*1, and f (Table 2).

IVIM has several advantages over traditional MR-perfusion method. A higher resolution could be achieved by IVIM. Meanwhile, IVIM is believed to be more sensitive. In addition, the parameters of IVIM are intrinsically quantitative, and they are sensitive to vasoconstriction and vasodilatation [24]. Finally, definition of tumor boundary on the basis of traditional MR based on contrast enhanced images is not successful in every patient.

IDH1 is believed to be the most frequent mutation site in glioma. Meanwhile, IDH1 mutations were also found in the anaplastic thyroid cancer, melanoma, acute myeloid leukemia patients [25]. IDH1 has been proved to be closely linked with oncogenesis through promoting DNA hypermethylation and stimulating hypoxia inducible factor-1 pathway [26]. However, several reports indicated that IDH1^*mut*^ patients had a longer survival time than IDH1^*wt*^ patients [27]. Therefore, the role of IDH1^*mut*^ in tumors have not been fully clarified.

There are some limitations in this study. Firstly, the sample size was relatively small, and only 78 patients were enrolled successfully in this research. Further large scale investigation needs to be conducted to confirm the conclusions of this study. Second, the quantification of CBF depends on performance of arterial input function.

In summary, IVIM-DWI presented efficacy in differentiating LGG and HGG patients. Meanwhile, IDH1^*mut*^ and IDH1^*wt*^ patients could be differentiated by parameters derived from IVIM-DWI.

## MATERIALS AND METHODS

### Patients

A total of 98 glioma patients diagnosed by MRI were enrolled from Oct 2018 to Oct 2020 in our hospital. The exclusion and inclusion criteria were listed in the Figure 1. After checking the examining materials, 20 patients were excluded due to incomplete pathological or imaging details. Finally, 78 gliomas patients confirmed by pathology data and IDH immunohistochemical results were included. Among them, 26 (16 males and 10 females) low grade gliomas (LGG, WHO grade I and grade II) cases were enrolled in this study. The age ranged from 14 to 65 years old (median age: 42 years). 10 cases were identified with IDH1 wild type and 16 cases were mutation type. 52 (30 males and 22 females) high grade gliomas (HGG, WHO grade III and grade IV) patients, aged 13-76 years (median age 55 years) were enrolled in this research. Among them, 41 cases were IDH1 wild type and 11 cases were mutant type.

All protocols in this study was approved by the Ethical Committee of The Second Affiliated Hospital of Nanchang University (Approval number: 2018-08-002), and was in accordance with the Helsinki declaration. Written informed consent was obtained from patients.

### Conventional MR imaging

Conventional MR imaging was conducted using a 3.0T MR scanner (Discovery MR 750; GE, Milwaukee, USA). The parameters were set as follows: T1-weighted (T1W) imaging, TI: 800 ms, TE: 25 ms, TR: 1900 ms. T2-weighted (T2W) imaging, TE: 100 ms, TR: 6600 ms, scanning layers: 24, layer spacing: 1 mm, layer thickness: 3 mm, matrix: 298×244, field of view: 240×240 mm.

### 3D pCASL

Firstly, the contrast agents were injected, then 3D pCASL was conducted. 3D FSE imaging sequences, background inhibition, and pseudo-continuous labeling were used in 3D pCASL. The parameters were set as follows: acquisition time: 5 min 16 s, NEX: 4, slices number: 40, thickness: 6 mm, bandwidth: ±60 kHz, field of view: 240×240 mm, post label delay: 1480 ms, TR/TE: 4686/8.

### IVIM-DWI

Firstly, the contrast agents were injected, then IVIM-DWI was conducted. A single-shot echo-planar sequence in the axial plane was applied for application of IVIM-DWI. The parameters were set as follows: matrix: 180×180, field of view: 240×240 mm, gap: 1 mm, thickness: 4 mm, TR/TE: 4500/6. 12 b values (0, 20, 40, 60, 90, 110, 150, 300, 600, 1200, 1800, and 2800 s/mm^2^) were applied during three orthogonal directions. The total acquisition time was 5 min 48 s.

### Imaging analysis

Images were moved to workstation (Advantage workstation, GE, Milwaukee, USA). Then, perfusion fraction (f), true diffusion coefficient (D), and perfusion-related diffusion coefficient (D*) were calculated according to the equation 1.

$${\text{S}}_{\left(\text{b}\right)}{\text{/S}}_{\left(0\right)}=\text{f}\cdot {\text{exp}}^{\left(-\text{b}\times \text{D}*\right)}+\left(1-\text{f}\right){\text{ exp}}^{\left(-\text{b}\times \text{D}\right)}$$ (1)

Fast moving compartment was represented by D*. The volume fraction of random microcirculation of blood over the total incoherent signal in each voxel was represented by f. The slow-moving compartment was represented by D. S_(b)_ and S_(0)_ represent the signal intensity in the presence and absence of diffusion sensitization, respectively. ADC was calculated with b values according to the equation 2. CBF maps were calculated as described [20].

$${\text{S}}_{\left(\text{b}\right)}{\text{/S}}_{\left(0\right)}={\text{exp}}^{\left(-\text{b}\times \text{D}\right)}$$ (2)

### Regions of interests (ROIs)

ROIs were conducted by the 3 experienced neuroradiologists. The maximum CBF values (CBF) of the tumor parenchyma areas (CBF1), peritumoral areas (CBF3) and mirror side normal areas (CBF2) were calculated. Then, relative CBF value (rCBF) was calculated as follows: rCBF = tumor side CBF value/mirror side CBF value. rCBF1-2= CBF1/CBF2, rCBF3-2= CBF3/CBF2.

Contrast-enhanced T2-FLAIR and T1WI was used to select the most obvious enhancement level or the largest level of tumor for analysis. The minimum D, maximum D * value, and maximum f value in tumor, peritumoral and normal areas were obtained on IVIM pseudo color image. D1, D*1, f1 were the IVIM parameters in the tumor parenchyma areas. D2, D*2, f2 were the IVIM parameters in mirror side normal areas. D3, D*3, f3 were the IVIM parameters in the peritumoral areas. Three ROIs (range 40-60 mm^2^) were drawn manually. Cerebrospinal fluid-filled, calcification, hemorrhagic, necrotic, and cystic areas should be avoided. The measurement was repeated twice with an interval of 1-2 weeks.

### Data analysis

Data analysis was performed using 21.0 SPSS (IBM, USA). Data was presented as mean ± standard deviation. The data between group HGG and LGG, group IDH1+ and IDH1- were analyzed using Student's t-test. Receiver operating characteristic curve (ROC) was performed to get the area under the receiver operating characteristic curve (AUC). The IVIM-DWI-derived parameters were analyzed using specificity, sensitivity, and predicted cutoff value from ROC. Intraclass coefficient (ICC) with the 95% interval confidence was set in this study. p<0.05 was believed to be statistical significance.

### Data availability

The data in the current study are available from the corresponding author on reasonable request.
